# The high volume of patients admitted during the SARS-CoV-2 pandemic has an independent harmful impact on in-hospital mortality from COVID-19

**DOI:** 10.1371/journal.pone.0246170

**Published:** 2021-01-28

**Authors:** Alessandro Soria, Stefania Galimberti, Giuseppe Lapadula, Francesca Visco, Agata Ardini, Maria Grazia Valsecchi, Paolo Bonfanti

**Affiliations:** 1 Clinic of Infectious Diseases, San Gerardo Hospital, School of Medicine and Surgery, University of Milano - Bicocca, Monza, Italy; 2 Bicocca Bioinformatics, Biostatistics and Bioimaging Center B4, School of Medicine and Surgery, University of Milano - Bicocca, Monza, Italy; 3 Medical Direction, Edoardo Bassini Hospital, Cinisello Balsamo, Italy; University of Sassari, ITALY

## Abstract

**Background:**

During the Coronavirus disease 2019 (COVID-19) pandemic, advanced health systems have come under pressure by the unprecedented high volume of patients needing urgent care. The impact on mortality of this “patients’ burden” has not been determined.

**Methods and findings:**

Through retrieval of administrative data from a large referral hospital of Northern Italy, we determined Aalen-Johansen cumulative incidence curves to describe the in-hospital mortality, stratified by fixed covariates. Age- and sex-adjusted Cox models were used to quantify the effect on mortality of variables deemed to reflect the stress on the hospital system, namely the time-dependent number of daily admissions and of total hospitalized patients, and the calendar period. Of the 1225 subjects hospitalized for COVID-19 between February 20 and May 13, 283 died (30-day mortality rate 24%) after a median follow-up of 14 days (interquartile range 5–19). Hospitalizations increased progressively until a peak of 465 subjects on March 26, then declined. The risk of death, adjusted for age and sex, increased for a higher number of daily admissions (adjusted hazard ratio [AHR] per an incremental daily admission of 10 patients: 1.13, 95% Confidence Intervals [CI] 1.05–1.22, p = 0.0014), and for a higher total number of hospitalized patients (AHR per an increase of 50 patients in the total number of hospitalized subjects: 1.11, 95%CI 1.04–1.17, p = 0.0004), while was lower for the calendar period after the peak (AHR 0.56, 95%CI 0.43–0.72, p<0.0001). A validation was conducted on a dataset from another hospital where 500 subjects were hospitalized for COVID-19 in the same period. Figures were consistent in terms of impact of daily admissions, daily census, and calendar period on in-hospital mortality.

**Conclusions:**

The pressure of a high volume of severely ill patients suffering from COVID-19 has a measurable independent impact on in-hospital mortality.

## Introduction

The Severe Acute Respiratory Syndrome Coronavirus 2 (SARS-CoV-2) pandemic has caused a disproportionate number of deaths worldwide, especially in some developed industrialized areas, despite well-equipped health systems. The concentration of many severely ill patients in a short period of time has almost overwhelmed hospital capacity. In Lombardy, Northern Italy, (83,820 cases and 15,296 deaths as of May 14, 2020), the rapid surge of cases of Coronavirus disease 2019 (COVID-19) requiring hospitalization has been managed by expanding bed capacity in intensive care units (ICU) and non- ICU wards [1–3].

The clinical hallmark of COVID-19 is interstitial pneumonia causing respiratory failure [4]. Initial symptoms, including both major (fever, cough, and dyspnea) and minor symptoms (alteration of the smell and taste, gastrointestinal symptoms, headache, and cutaneous manifestations), are poorly predictive of subsequent severe evolution, thus challenging the triage for hospitalization [5–8].

The high observed in-hospital mortality was attributed, among other factors, to the large number of cases admitted within a short time period, stressing hospital system capacity [9, 10]. However, this assumption has not been proven. The net contribution of patients’ burden in determining mortality has been poorly explored so far.

The aim of this study was to assess the relationship between daily hospital admissions, daily hospital census, and calendar date on in-hospital mortality in a large referral hospital that rapidly increased bed capacity during the COVID-19 outbreak in March 2020.

## Methods

Data on sex, age, day of admission/discharge and status were retrieved from administrative records during the study period (from February 20 to May 13, 2020). Aalen-Johansen cumulative incidence curves were used to describe in-hospital mortality stratified by fixed covariates, with discharge as competing event, and the Gray test was used for comparison. Age (<65 *versus* 65–75 *versus* >75 years)- and sex-adjusted Cox models were used to quantify the effect on mortality of variables indicating stress on the hospital system: the time-dependent daily number of admissions, total hospitalized census, and calendar period (before *versus* after the peak of the epidemic of March 26).

To appraise generalizability, we conducted a validation analysis on a same-structured administrative dataset coming from another hospital which faced the same rapid surge of COVID-19 patients in the same period and behaved accordingly by expanding bed capacity.

Statistical analyses were performed using SAS software (version 9.4) and R software (version 3.6.0).

### Ethics statement

Data analysis was approved by local Institutional Boards (Medical Direction San Gerardo Hospital and Medical Direction Bassini Hospital). As anonymized data came from administrative databases fueled by daily hospital census for management purposes, given the nature of the study, written informed consent was not required.

## Results

### Main analysis on San Gerardo Hospital

**Fig 1:**
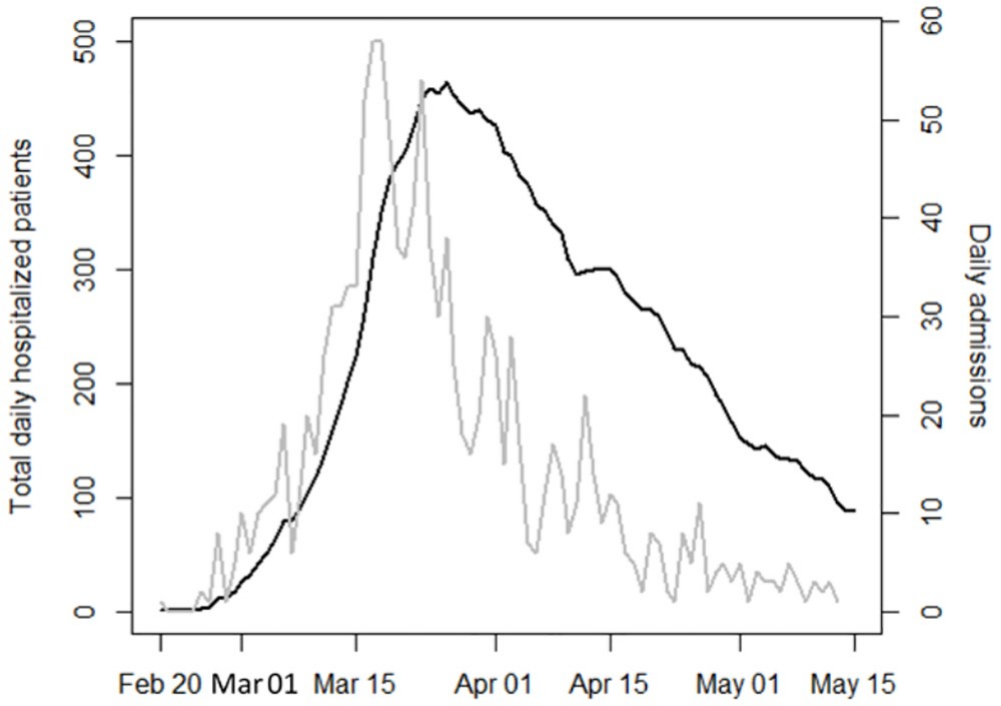
Distribution of inpatients over time at San Gerardo Hospital. The number of daily admissions (gray) increased progressively until a peak of 58 patients on March 18, and the number of total hospitalized patients (black) peaked on March 26, with 465 subjects.

**Fig 2:**
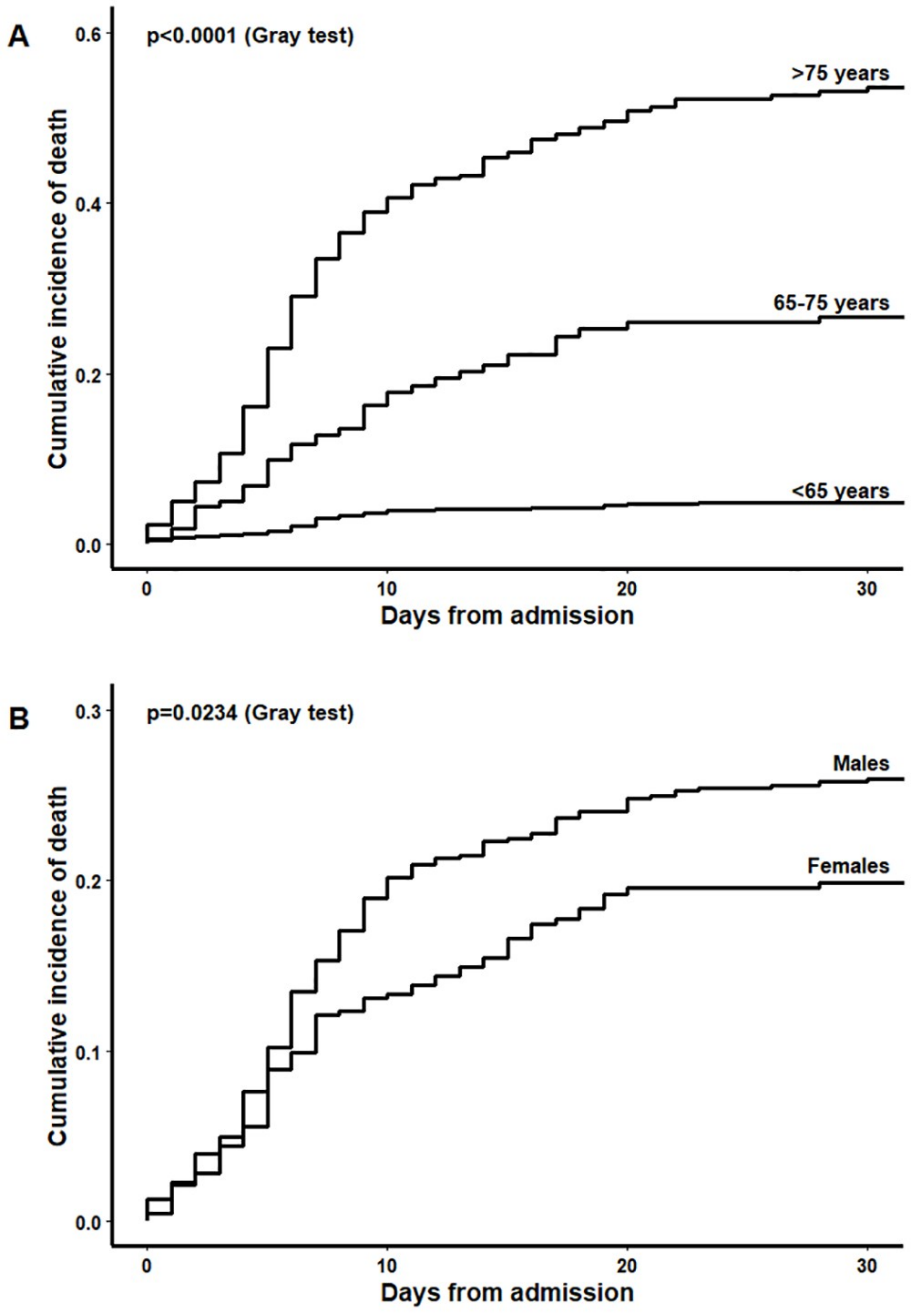
Crude-incidence curves of in-hospital mortality at San Gerardo Hospital, stratified by age strata (A) and sex (B).

**Fig 3:**
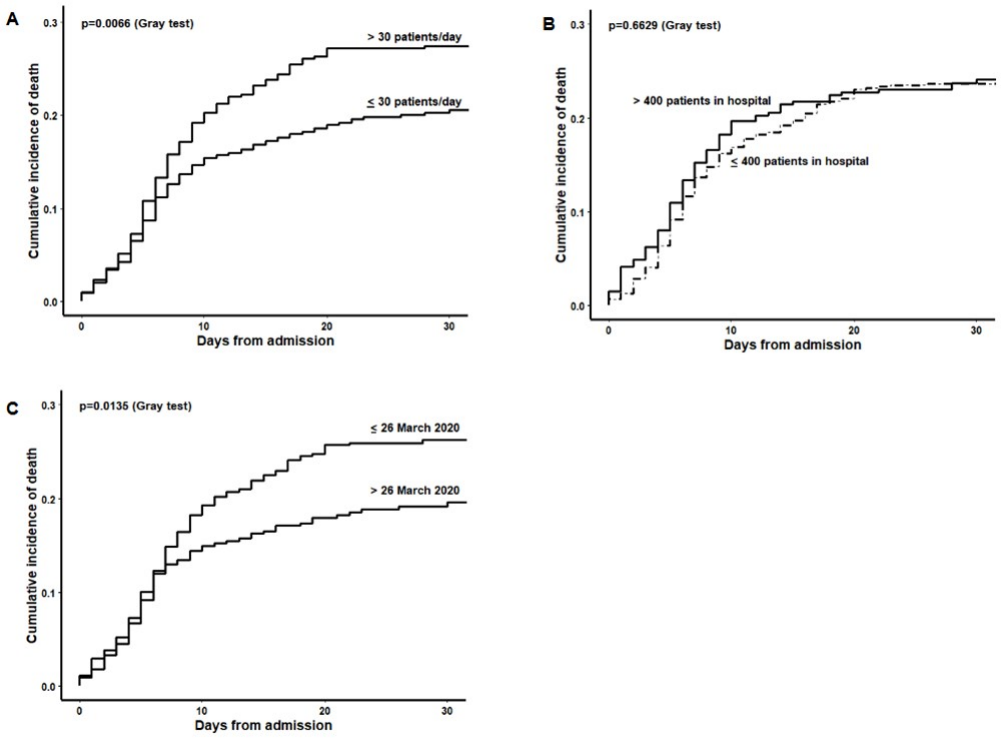
Crude-incidence curves of in-hospital mortality at San Gerardo Hospital, stratified by “hospital stress” variables. Variables of “hospital stress” are considered as fixed variables measured at patient hospital admission. A: number of daily admissions; B: total daily census; C: calendar period before or after the peak.

Between February 20 and May 13, 1225 subjects (32.8% females, mean age ±standard deviation [SD] 65.5 ±15.0 years, 65.4 ±14.0 years in males and 65.8 ±16.5 years in females) were admitted with a diagnosis of COVID-19 in San Gerardo Hospital, Monza, Italy. Hospital admissions increased progressively, rising to a peak of 465 total hospitalized patients on March 26, and slowly declining thereafter (Fig 1). Sex and age distribution by calendar period showed an increase of females (from 31.2% to 42.9%) and elderly patients (>75 years: from 25.5% to 36.5%) after the peak (Table 1). After a median follow-up of 14 days (1^st^-3^rd^ quartiles 5–19), 283 in-hospital deaths occurred (30-day mortality rate 24%, higher in the elderly and in males, Table 2, Fig 2A and 2B). The crude-incidence curves of in-hospital mortality highlight the different outcomes according to different indicators of “hospital pressure” at patient admission, considered as fixed variables (Fig 3A–3C).

**Table 1:** Age and sex distribution of patients hospitalized at San Gerardo Hospital.

	February 20-March 26			March 27- May 13		
Age, y	M	F	Total	M	F	Total
**<65**	275 (51.1)	117 (47.9)	392 (50.1)	113 (44.7)	80 (42.1)	193 (43.6)
**65–75**	123 (22.9)	68 (27.9)	191 (24.4)	59 (23.3)	29 (15.3)	88 (19.9)
**>75**	140 (26.0)	59 (24.2)	199 (25.5)	81 (32.0)	81 (42.6)	162 (36.5)
**Total**	538 (100)	244 (100)	782 (100)	253 (100)	190 (100)	443 (100)

Age and sex distribution of patients admitted to San Gerardo Hospital between February 20 and May 13, 2020, divided according to the peak of the epidemic, which occurred on March 26. Values are expressed as numbers and percentages (in parentheses). M = males; F = females; y = years. Differences in the distribution of age and sex in the two periods are statistically significant (Chi-square p <0.001).

**Table 2:** Mortality according to age, sex, and variables of “hospital stress”.

Characteristic	n. deaths	n. patients	rate (%)
**Age (years)**			
**<65**	33	585	(5.6)
**65–75**	74	279	(26.5)
**>75**	176	361	(48.8)
**Sex**			
**M**	201	791	(25.4)
**F**	82	434	(18.9)
**Patients daily admissions**			
**≤30**	124	638	(19.4)
**>30**	159	587	(27.1)
**Total hospitalized patients**			
**≤400**	191	839	(22.8)
**>400**	92	386	(23.8)
**Calendar period**			
**February 20-March 26**	202	782	(25.8)
**March 27- May 13**	81	443	(18.3)
**Total**	283	1225	(23.1)

Rate of death of patients hospitalized at San Gerardo Hospital, according to age, sex, number of daily admissions, total number of hospitalized patients measured at admission, and calendar period. M = males; F = females.

**Fig 4:**
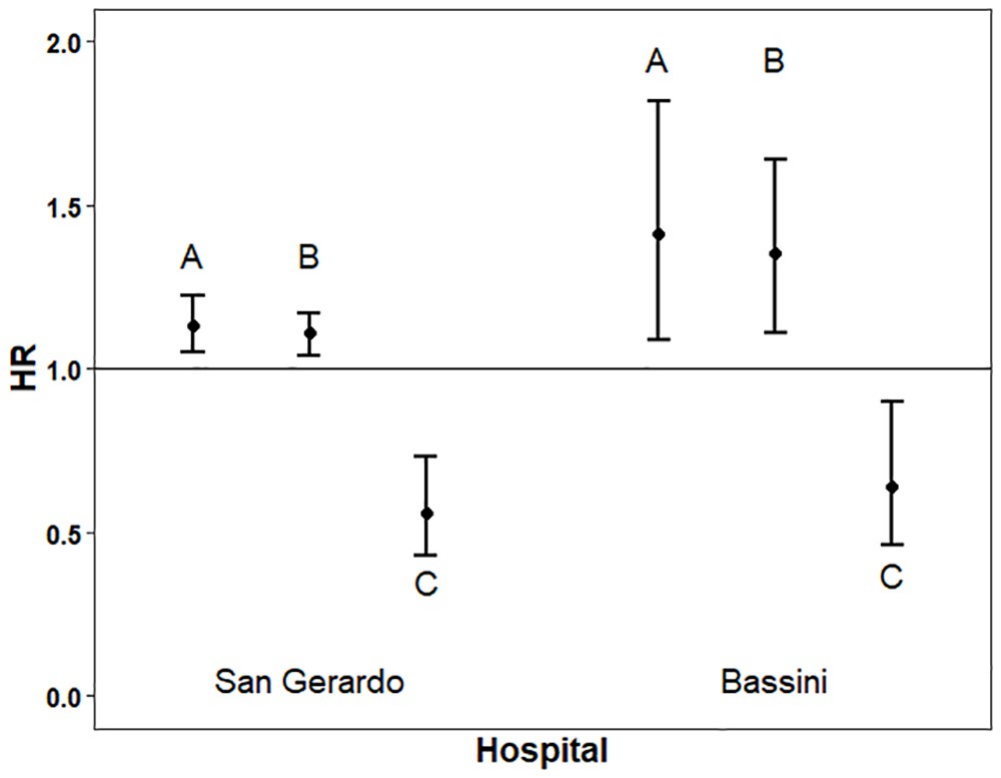
Risk of death in the time-dependent Cox model by hospital stress variables. Circles and bars represent the age- and sex-adjusted hazard ratios (HR) and the corresponding 95% Confidence Intervals of death, according to A) daily number of admissions (per increase of 10 units), B) total daily census (per increase of 50 units, C) calendar period (after *versus* before the peak). On the left: San Gerardo Hospital. On the right: Bassini Hospital.

In the time-dependent Cox models, we assessed the individual effect of each of three variables deemed to reflect the influence of patient load on mortality. The adjusted hazard ratios were: 1.13 (95% Confidence Intervals [CI] 1.05–1.22, p = 0.0014) for an incremental daily admission of 10 patients, 1.11 (95%CI 1.04–1.17, p = 0.0004) for an increase of 50 patients in the total number of hospitalized subjects, and 0.56 (95%CI 0.43–0.73, p<0.001) for the calendar period after *versus* before the peak (Fig 4).

Older age and male sex were significantly related to mortality.

### Validation analysis on Bassini Hospital

In the same time-period, 500 patients (36.4% females, mean ±SD age 70.0 ±15.6 years, 68.8 ±15.1 years in males and 72.0 ±16.1 years in females) were admitted with a diagnosis of COVID-19 in Bassini Hospital, Cinisello Balsamo, Italy. Hospital admissions increased progressively, rising to a peak of 178 total hospitalized patients on March 26, and slowly declining thereafter (S1 Fig). Age and sex distribution before and after the peak is shown in S1 Table. After a median follow-up of 16 days (1^st^-3^rd^ quartiles 10–25), 161 in-hospital deaths occurred (30-day mortality rate 31%, higher in the elderly and in males, S2 Table, S2A and S2B Fig). The crude-incidence curves of in-hospital mortality highlight the different outcomes according to different indicators of “hospital pressure” at patient admission, considered as fixed variables (S3A–S3C Fig).

In the time-dependent Cox models to assess the variables of hospital stress, the corresponding adjusted hazard ratios were: 1.41 (95%CI 1.09–1.82, p = 0.0084) for an incremental daily admission of 10 patients, 1.35 (95%CI 1.11–1.64, p = 0.0025) for an increase of 50 patients in the total number of hospitalized subjects, and 0.64 (95%CI 0.46–0.90, p = 0.0108) for the calendar period after *versus* before the peak (Fig 4).

## Discussion

In a large dataset of patients hospitalized for COVID-19, we showed an impact of the volume of hospital admissions on the risk of death, suggesting that pressure on a saturated health system had a measurable effect on mortality. This was observed for both daily admissions and total daily census, considered as time-dependent variables in the Cox models, thus accounting for the “hospital stress” along all the period of observation. In addition, there was a marked decrease in mortality by calendar period, possibly reflecting the decreased pressure on the hospital, as well as a learning-curve effect. The adaptive response of the hospital by rapidly increasing bed capacity was overwhelmed by the sharp rise in patients arriving simultaneously, suggesting issues in staff preparedness, oxygen supply, and devices availability, which were then mitigated possibly by the increased clinical/organizational experience and a progressive decrease of admissions.

Our findings were corroborated by the validation on another dataset coming from a different hospital which faced the same surge of COVID-19 patients during the same time period. In this setting too, the number of daily admissions, the total daily census, and the calendar period before the peak had a significant impact on the subsequent risk of in-hospital death. The validation on another dataset provided results that go in the same direction, thus adding consistency to our original findings.

The impact of hospital overcrowding on patient outcome has already been described in different settings [11–13]. In China, differences in “COVID-19 burden” on healthcare system across provinces has been associated with different mortality [14]. In the recent COVID-19 outbreak in Lombardy, Italy, the approach of daily expanding bed capacity to face the rapid surge of critical cases was not sufficient to cope with the impending number of incoming patients, generating a “tsunami effect”. In this study, we suggest an easy tool to possibly measure the impact of this impressive surge of hospitalizations on subsequent patients’ outcome.

From a public health perspective, our data emphasize the importance of policies aimed at avoiding an acute surge in hospitalizations for COVID-19. Our findings suggest the need for better equipped emergency rooms (space, staff, oxygen devices, etc.), and more rapid deployment of expanded medical wards in future epidemics [15–17].

This study has limitations: as it is based solely on administrative records, it does not account for co-morbidities, which could have influenced the final outcome. However, in the literature on COVID-19, age seems to be the major contributor to the risk of death [4, 18]. Our data confirm this trend, so that age could be considered a good proxy for co-morbidities (especially hypertension and cardiovascular disease, the most frequent conditions associated with higher mortality from COVID-19). Moreover, it is implausible that patients with different co-morbidities distributed differently over time. As we have adjusted for age and sex, our findings seem consistent across different types of patients. A second limitation is that the variables considered as indicators of “hospital stress” could not take into account many interfering factors, such as the heterogeneity of newly created COVID-19 wards, especially in terms of staff preparedness. However, the consistency of different easy-to-measure stress indicators highlights a role of patients’ concentration in influencing the risk of death. Basing our analysis on administrative data, we could not control for clinical severity of COVID-19 at admission. Still, the capacity of our hospital increased progressively to face the surge of cases requiring hospitalization, with the mantra of "no patient left behind": many ICU beds and entire non-ICU wards were opened overnight, physicians and nurses doubled their shifts for two months. In other words, admission criteria did not change overtime, as capacity adapted quickly to the increasing number of patients. Moreover, the Cox analysis to estimate the risk of death considered the stress variables as time-dependent, meaning that the effect of patient overload is considered daily from admission to the event (death or discharge, considered as competing events), thus attenuating the possible bias of abnormal concentration of severe cases in the very same days of the peak of the epidemic (which cannot be excluded, but is not proven).

Notwithstanding these limitations, we were able to show a measurable impact of patients’ burden on 30-day in-hospital mortality by defining variables which reflect the pressure of impending continuous patients’ flow on the hospital system, and calculating the age- and sex-adjusted hazard ratios of death for these “stress indicators”. The validation of this model on another dataset accrues consistency to our findings.

Current knowledge on the best management of mass emergency, as was COVID-19 during the peak of the outbreak, is focused in rapidly scaling-up bed capacity and intensive care resources to cope with the surge of critical cases. Our study, which identified an independent risk of death in patients’ burden *per se*, put the accent on the necessity of rethinking the entire process of patients’ management, both through public health measure to flatten the epidemic curve, and by better preparing to receive the wave of critically-ill patients in the hospital. Ascertaining pressure to the hospital system through defined “stress variables” could be a valuable tool to predict the impact of patients’ burden and prepare the health system accordingly.

As the COVID-19 pandemic is far from over, we believe that our measurement of the impact of patients’ burden on in-hospital mortality, which has never been shown so far, could set up the basis for future implementation research on hospital care.

## Supporting information

S1 Table Age and sex distribution of patients hospitalized at Bassini Hospital. (DOCX)

S2 Table Mortality according to age, sex, and variables of “hospital stress”. (DOCX)

S1 Fig Distribution of inpatients over time at Bassini Hospital. (DOCX)

S2 Fig Crude-incidence curves of in-hospital mortality at Bassini Hospital, stratified by age strata (A) and sex (B). (DOCX)

S3 Fig Crude-incidence curves of in-hospital mortality at Bassini Hospital, stratified by “hospital stress” variables. (DOCX)

S1 Data (XLSX)

## References

[1] Grasselli G, Pesenti A, Cecconi M. Critical Care Utilization for the COVID-19 Outbreak in Lombardy, Italy: Early Experience and Forecast During an Emergency Response. JAMA, 2020; 323: 1545–1546. 10.1001/jama.2020.4031 32167538

[2] Remuzzi A, Remuzzi G. COVID-19 and Italy: what next?. Lancet, 2020; 395: 1225–1228. 10.1016/S0140-6736(20)30627-9 32178769 PMC7102589

[3] Grasselli G, Zangrillo A, Zanella A, et al Baseline Characteristics and Outcomes of 1591 Patients Infected With SARS-CoV-2 Admitted to ICUs of the Lombardy Region, Italy. JAMA, 2020; 323: 1574–1581. 10.1001/jama.2020.5394 32250385 PMC7136855

[4] Zhou F, Yu T, Du R, et al Clinical course and risk factors for mortality of adult inpatients with COVID-19 in Wuhan, China: a retrospective cohort study. Lancet 2020;395:1054–1062. 10.1016/S0140-6736(20)30566-3 32171076 PMC7270627

[5] Wang D, Hu B, Hu C, et al Clinical characteristics of 138 hospitalized patients with 2019 novel coronavirus-infected pneumonia in Wuhan, China. JAMA 2020;323:1061–1069. 10.1001/jama.2020.1585 32031570 PMC7042881

[6] Pan L, Mu M, Yang P, et al Clinical characteristics of COVID-19 patients with digestive symptoms in Hubei, China: a descriptive, cross-sectional, multicenter study. Am J Gastroenterol 2020;115:766–773. 10.14309/ajg.0000000000000620 32287140 PMC7172492

[7] Meng X, Deng Y, Dai Z, Meng Z. COVID-19 and anosmia: a review based on up-to-date knowledge. Am J Otolaryngol 2020;41:102581–102581. 10.1016/j.amjoto.2020.102581 32563019 PMC7265845

[8] Huang C, Wang Y, Li X, et al Clinical features of patients infected with 2019 novel coronavirus in Wuhan, China. Lancet 2020;395:497–506. 10.1016/S0140-6736(20)30183-5 31986264 PMC7159299

[9] Odone A, Delmonte D, Scognamiglio T, Signorelli C. COVID-19 deaths in Lombardy, Italy: data in context. Lancet Public Health, 2020; 5: e310 Erratum in: Lancet Public Health, 2020; 5: e315. 10.1016/S2468-2667(20)30099-2 32339478 PMC7182509

[10] Zangrillo A, Gattinoni L. Learning from mistakes during the pandemic: the Lombardy lesson. Intensive Care Med, 2020; 46: 1622–1623. 10.1007/s00134-020-06137-9 32504102 PMC7272593

[11] Sun BC, Hsia RY, Weiss RE, et al Effect of emergency department crowding on outcomes of admitted patients. Ann Emerg Med, 2013; 61: 605–611. 10.1016/j.annemergmed.2012.10.026 23218508 PMC3690784

[12] Jo S, Kim K, Lee JH, et al Emergency department crowding is associated with 28-day mortality in community-acquired pneumonia patients. J Infect, 2012; 64: 268–275. 10.1016/j.jinf.2011.12.007 22227383

[13] Berg LM, Ehrenberg A, Florin J, Östergren J, Discacciati A, Göransson KE. Associations Between Crowding and Ten-Day Mortality Among Patients Allocated Lower Triage Acuity Levels Without Need of Acute Hospital Care on Departure From the Emergency Department. Ann Emerg Med, 2019; 74: 345–356. 10.1016/j.annemergmed.2019.04.012 31229391

[14] Ji Y, Ma Z, Peppelenbosch MP, Pan Q. Potential association between COVID-19 mortality and health-care resource availability. Lancet Glob Health, 2020; 8: e480 10.1016/S2214-109X(20)30068-1 32109372 PMC7128131

[15] Adhikari NK, Fowler RA, Bhagwanjee S, Rubenfeld GD. Critical care and the global burden of critical illness in adults. Lancet, 2010; 376: 1339–1346. 10.1016/S0140-6736(10)60446-1 20934212 PMC7136988

[16] Carenzo L, Costantini E, Greco M, et al Hospital surge capacity in a tertiary emergency referral centre during the COVID-19 outbreak in Italy. Anaesthesia, 2020; 75: 928–934. 10.1111/anae.15072 32246838

[17] Fagiuoli S, Lorini FL, Remuzzi G; Covid-19 Bergamo Hospital Crisis Unit. Adaptations and Lessons in the Province of Bergamo. N Engl J Med, 2020; 382: e71 10.1056/NEJMc2011599 32369276 PMC7219535

[18] Petrilli CM, Jones SA, Yang J, et al Factors associated with hospital admission and critical illness among 5279 people with coronavirus disease 2019 in New York City: prospective cohort study. BMJ, 2020; 369: m1966 10.1136/bmj.m1966 32444366 PMC7243801

