# Supplementary material for: The high volume of patients admitted during the SARS-CoV-2 pandemic has an independent harmful impact on in-hospital mortality from COVID-19
Source: PLoS One. 2021 Jan 28;16(1):e0246170. doi: 10.1371/journal.pone.0246170 (PMC7842950; doi:10.1371/journal.pone.0246170)
Supplement: S1 Table — (DOCX) [file pone.0246170.s001.docx]

**S1 Table. Age and sex distribution of patients hospitalized at Bassini Hospital**

|  | **February 20-March 26** | | | **March 27- May 13** | | |
| --- | --- | --- | --- | --- | --- | --- |
| **Age, y** | **M** | **F** | **Total** | **M** | **F** | **Total** |
| **<65** | 91 (43.1) | 30 (29.7) | 121 (38.8) | 42 (39.3) | 29 (35.8) | 71 (37.8) |
| **65-75** | 46 (21.8) | 15 (14.9) | 61 (19.6) | 10 (9.4) | 10 (12.4) | 20 (10.6) |
| **>75** | 74 (35.1) | 56 (55.5) | 130 (41.7) | 55 (51.4) | 42 (51.9) | 97 (51.6) |
| **Total** | 211 (100) | 101 (100) | 312 (100) | 107 (100) | 81 (100) | 188 (100) |

Age and sex distribution of patients admitted to Bassini Hospital between February 20 and May 13, 2020, divided according to the peak of the epidemic, which occurred on March 26. Values are expressed as numbers and percentages (in parentheses). M = males; F = females; y = years. Differences in the distribution of age and sex in the two periods are statistically significant (Chi-square p <0.001).
