# Supplementary material for: The high volume of patients admitted during the SARS-CoV-2 pandemic has an independent harmful impact on in-hospital mortality from COVID-19
Source: PLoS One. 2021 Jan 28;16(1):e0246170. doi: 10.1371/journal.pone.0246170 (PMC7842950; doi:10.1371/journal.pone.0246170)
Supplement: S2 Table — (DOCX) [file pone.0246170.s002.docx]

**S2 Table. Mortality according to age, sex, and variables of “hospital stress”**

| **Characteristic** | **n. deaths** | **n. patients** | **rate (%)** |
| --- | --- | --- | --- |
| **Age (years)** |  |  |  |
| **<65** | 15 | 192 | (7.8) |
| **65-75** | 29 | 81 | (35.8) |
| **>75** | 117 | 227 | (51.5) |
| **Sex** |  |  |  |
| **M** | 111 | 318 | (34.9) |
| **F** | 50 | 182 | (27.5) |
| **Patients daily admissions** |  |  |  |
| **≤10** | 64 | 217 | (29.4) |
| **>10** | 97 | 283 | (34.3) |
| **Total hospitalized patients** |  |  |  |
| **≤150** | 95 | 303 | (31.3) |
| **>150** | 66 | 197 | (33.5) |
| **Calendar period** |  |  |  |
| **February 20-March 26** | 112 | 312 | (35.9) |
| **March 27- May 13** | 49 | 188 | (26.1) |
| **Total** | 161 | 500 | (32.2) |

Rate of death of patients hospitalized at Bassini Hospital, according to age, sex, number of daily admissions, total number of hospitalized patients measured at admission, and calendar period.

M = males; F = females.
