# Supplementary material for: The high volume of patients admitted during the SARS-CoV-2 pandemic has an independent harmful impact on in-hospital mortality from COVID-19
Source: PLoS One. 2021 Jan 28;16(1):e0246170. doi: 10.1371/journal.pone.0246170 (PMC7842950; doi:10.1371/journal.pone.0246170)

**S1 Fig. Distribution of inpatients over time at Bassini Hospital**

The number of daily admissions (gray) increased progressively until a peak of 26 patients on March 14, and the number of total hospitalized patients (black) peaked on March 26, with 178 subjects.


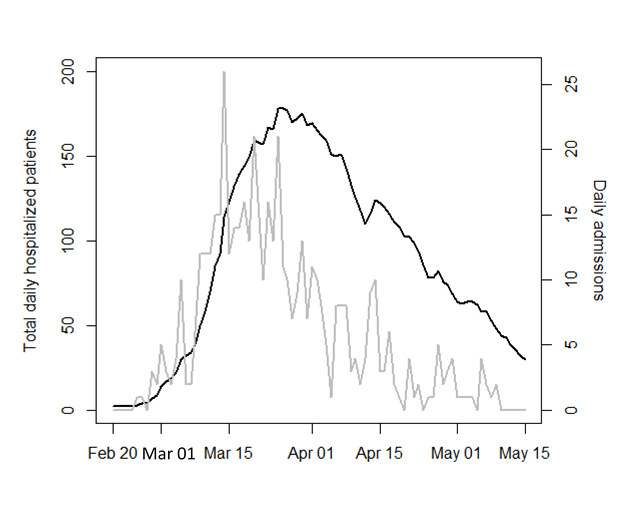

Supplement: S1 Fig — (DOCX) [file pone.0246170.s003.docx]
