# Supplementary material for: The high volume of patients admitted during the SARS-CoV-2 pandemic has an independent harmful impact on in-hospital mortality from COVID-19
Source: PLoS One. 2021 Jan 28;16(1):e0246170. doi: 10.1371/journal.pone.0246170 (PMC7842950; doi:10.1371/journal.pone.0246170)

**S2 Fig. Crude-incidence curves of in-hospital mortality at Bassini Hospital, stratified by age strata (A) and sex (B)**

**A**


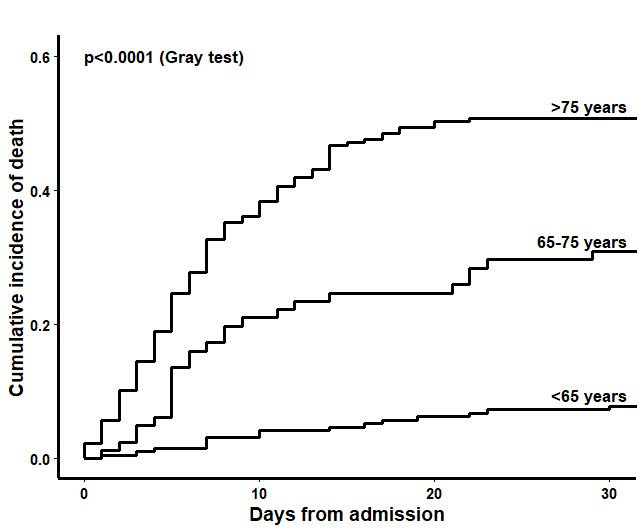


**B**


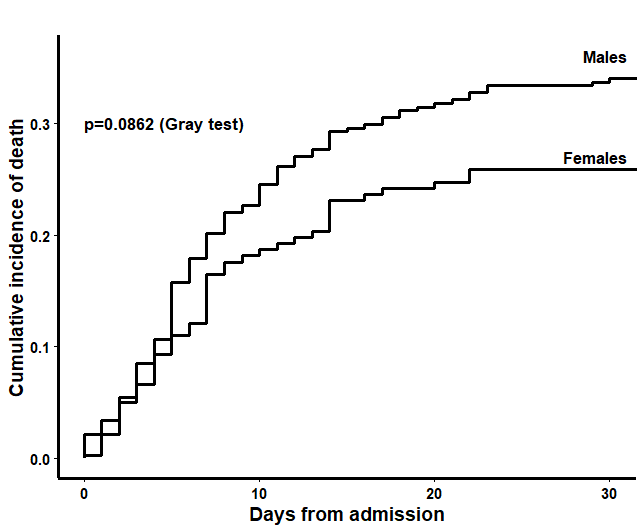

Supplement: S2 Fig — (DOCX) [file pone.0246170.s004.docx]
